# 3D Printed Wavy Scaffolds Enhance Mesenchymal Stem Cell Osteogenesis

**DOI:** 10.3390/mi11010031

**Published:** 2019-12-25

**Authors:** Shen Ji, Murat Guvendiren

**Affiliations:** 1 Otto H. York Department of Chemical and Materials Engineering, New Jersey Institute of Technology, University Heights, Newark, NJ 07102, USA; SJ422@njit.edu; 2 Department of Biomedical Engineering, New Jersey Institute of Technology, University Heights, Newark, NJ 07102, USA

**Keywords:** biomaterials, additive manufacturing, stem cells, tissue engineering, bone regeneration

## Abstract

There is a growing interest in developing 3D porous scaffolds with tunable architectures for bone tissue engineering. Surface topography has been shown to control stem cell behavior including differentiation. In this study, we printed 3D porous scaffolds with wavy or linear patterns to investigate the effect of wavy scaffold architecture on human mesenchymal stem cell (hMSC) osteogenesis. Five distinct wavy scaffolds were designed using sinusoidal waveforms with varying wavelengths and amplitudes, and orthogonal scaffolds were designed using linear patterns. We found that hMSCs attached to wavy patterns, spread by taking the shape of the curvatures presented by the wavy patterns, exhibited an elongated shape and mature focal adhesion points, and differentiated into the osteogenic lineage. When compared to orthogonal scaffolds, hMSCs on wavy scaffolds showed significantly enhanced osteogenesis, indicated by higher calcium deposition, alkaline phosphatase activity, and osteocalcin staining. This study aids in the development of 3D scaffolds with novel architectures to direct stem osteogenesis for bone tissue engineering.

## 1. Introduction

There is a growing interest in developing porous scaffolds for bone tissue engineering enabling temporary mechanical support for cells to attach, migrate, and produce newly formed extracellular matrix to form functional bone tissue ultimately [1,2,3]. Although bone has a robust regenerative ability, therapeutic interventions are required for large bone defects [4,5]. Grafts (autografts, allografts, and xenografts) are commonly used in the clinic to fill the defect site and to regenerate bone tissue [6,7]. Porous scaffolds can be considered as an alternative to regenerate bone while mechanically supporting the defect site [8]. A wide range of techniques have been developed to fabricate porous bone scaffolds, such as gas foaming [9,10,11], solvent casting and particle/salt leaching [12,13,14,15,16], phase separation [17,18], freeze drying [19,20], and electrospinning [21,22,23]. However, the majority of these techniques fail to control the 3D architecture of the scaffolds precisely, including pore size and pore distribution, and also fail to develop reproducible scaffolds [1]. 3D printing is an additive manufacturing technique and enables the fabrication of custom designed and highly complex 3D scaffolds. 3D printing allows the use of the patient’s own medical images to design personalized scaffolds that are anatomically similar to the defect site. Thus, it has been widely utilized for fabricating custom designed bone scaffolds [24,25,26,27,28]. A wide range of 3D printing techniques have been used to fabricate 3D bone scaffolds, such as fused deposition modeling (FDM) [29,30,31,32], direct ink writing (DIW) [33,34], selective laser sintering and melting (SLS and SLM) [35], stereolithography (SLA) [36,37,38], continuous digital light processing (cDLP) [39,40], and inkjet printing [41,42]. These 3D printing technologies allow utilizing various printable materials [43] and designs [44]. Computational tools have also been utilized to optimize scaffold architecture to achieve enhanced permeability and mechanical properties [45,46,47,48,49]. 

Mesenchymal stem cells (MSCs) are regarded as a clinically relevant cell source for bone tissue engineering due to their ability to proliferate and migrate, as well as their potential to differentiate into the osteogenic lineage (bone) [50,51,52,53]. Stem cells are known to feel and respond to their microenvironment by regulating their function [54,55,56,57]. Materials based approaches have been developed to engineer extracellular matrix (ECM) mimetic microenvironments [58,59,60], including macro- and nano-scale topographical cues to control stem cell behavior [61,62]. Topographical cues alone have been shown to control stem cell response, such as morphology, alignment, proliferation, migration, cytoskeletal organization, focal adhesion, nuclear deformation, and differentiation [62,63,64]. For example, human MSCs (hMSCs) are shown to produce bone mineral when cultured on substrates with the nanoscale order [65]. Nanoscale roughness is shown to enhance MSC osteogenesis even in the absence of induction media [66,67]. This phenomenon is shown to be due to clustering of absorbed proteins on a nano-topography, which promotes integrin mediated focal adhesions, enhancing cellular contractility and stem cell osteogenesis [66]. Microscale patterns confining stem cells within cell adhesive regions were used to control stem cell shape or cellular spreading. For instance, McBeath et al. showed that hMSCs with the spread morphology led to actin-myosin generated tension and promoted osteogenic differentiation [68]. Increasing cellular contractility, or cytoskeletal tension, by changing the shape of the multicellular sheets, Ruiz and Chen were able to enhance osteogenic differentiation of hMSCs [69]. Mrksich and co-workers showed that stem cells residing on curved surfaces became highly contractile and differentiated to the osteogenic lineage [70]. Lineage commitment of hMSCs on hydrogel wrinkling patterns was determined by the pattern morphology, such that hMSCs on lamellar patterns formed a spread morphology with a high cell aspect ratio (>4) differentiated into osteogenic progenitors [71]. When porous 3D scaffolds are considered, pore architecture, surface topography, and interconnectivity are shown to control osteogenic differentiation of human mesenchymal progenitor cells [72]. Simon and co-workers fabricated 2D films and 3D porous scaffolds with different techniques (gas foaming, salt leaching, phase separation, electrospinning, 3D printing, and spin coating) to examine the seeded hMSCs’ osteogenesis, which indicated that the scaffolds could be optimized to control the cell morphology to direct differentiation [73]. Recently, DIW was used to create 3D scaffolds with distinct architectures composed of square (SQR), hexagonal (HEX), or octagonal (OCT) patterns [74]. Human MSCs were reported to exhibit a higher cell aspect ratio and mean cell area on OCT scaffolds as compared to SQR and HEX scaffolds, and hence showed significantly enhanced osteogenic differentiation. Although the effect of curvature is well documented in 2D, it has not yet been studied systematically in 3D.

In this work, we used 3D printing to fabricate wavy poly(caprolactone) (PCL) scaffolds to investigate the effect of curvature on hMSC osteogenesis. A sinusoidal waveform was used to create wavy scaffolds. The wavelength and amplitude of the sinusoid were systematically varied to design five distinct wavy scaffolds. An orthogonal scaffold with straight struts was used as a control. First, we investigated the effects of scaffold architecture on stem cell growth, including cell attachment, proliferation, and shape (spreading). Then, we studied the osteogenic differentiation of hMSCs on wavy scaffolds as compared to the commonly used orthogonal architecture. The main hypothesis behind this study was that the wavy scaffolds can direct a more elongated and stretched stem cell morphology, resulting in highly organized cytoskeletal arrangement with high contractility. This could lead to an increased osteogenesis, the degree of which can be controlled by the degree of the curvature or waviness.

## 2. Materials and Methods

### 2.1. Scaffold Design

**Figure 1:**
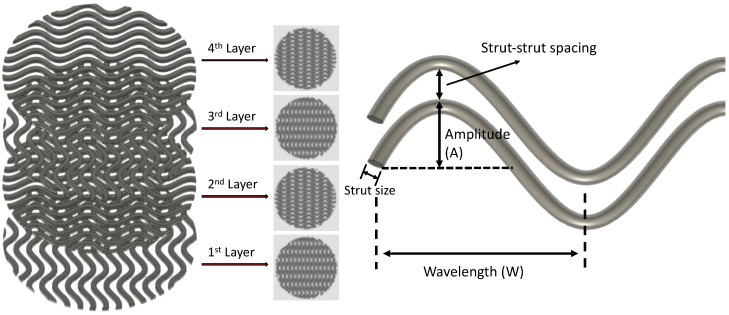
Wavy scaffold design containing 4 layers (**left**) and schematic showing the strut design for wavy scaffolds (**right**).

Autodesk^®^ Fusion 360™ (Autodesk Inc., San Rafael, CA, USA) was used to design the 3D models. The basic 3D model was designed as a cylinder with a diameter of 15 mm and a height of 1 mm. The 3D model (.stl file) was then loaded into Perfactory RP for slicing, with a layer height equal to 0.25 mm. The sliced file (.bpl file) was loaded into Visual Machine, and the infill patterns were selected. A linear pattern was selected for the orthogonal scaffolds (i.e., the control group), and a sinusoidal waveform was selected for the wavy scaffolds (Figure 1). For wavy scaffolds, the amplitude and the wavelength of the sinusoid were varied systematically to develop 5 distinct scaffold designs (Table 1).

### 2.2. 3D Printing of Scaffolds

3D Bioplotter (EnvisionTEC, Gladbeck, Germany) was used to print the scaffolds using PCL pellets (MW = 55 kDa, Polysciences Inc., Warrington, PA, USA). The print temperature and pressure were set to 80 °C and 700 kPa (7 bar), whereas the print speed was varied from 4 to 6 mm/s for each design to achieve a similar strut size (see Table 1 for actual values for each design).

### 2.3. Characterization of the Scaffolds

3D printed scaffolds were imaged by using a scanning electron microscope (SEM, JSM-7900F, JEOL, Tokyo, Japan) and a micro-computed tomography scanner (micro-CT, SkyScan 1275, Bruker, Billerica, MA, USA). SEM images were used to measure the strut size and the strut-to-strut distance. Micro-CT was used to measure the porosity of the scaffolds. Compression tests were performed on 3D printed scaffolds using an Instron machine (model 3343) with a 1000 N load cell and a 0.5 mm/min displacement rate. Three samples for each scaffold group were tested.

### 2.4. Preparation of the Scaffolds for Cell Culture

Scaffolds were sterilized by immersing them in a 75% ethanol solution for 30 min, followed by 1 h of ultraviolet (UV) light exposure (by a germicidal lamp) for each side of the scaffold. Scaffolds were then incubated in 300 μL of fibronectin solution (20 μg/mL, bovine fibronectin plasma, Invitrogen) overnight to enhance cell attachment. Fibronectin solution was removed, and scaffolds were washed with Dulbecco’s Phosphate Buffered Saline (DPBS, Gibco, New York, NY, USA). Scaffolds were then moved into a new well and kept in growth media prior to cell seeding.

### 2.5. Cell Culture and Reagents

Human mesenchymal stem cells (hMSCs, passage 4, Lonza, Walkersville, MD, USA) were cultured in growth media (α-MEM (minimum essential medium) supplemented with 10% fetal bovine serum (FBS, Gibco, New York, NY, USA) and 1% penicillin-streptomycin (pen-strep, Gibco, New York, NY, USA)). Prior to seeding, each scaffold was removed from the growth media and placed in a single well in a non-treated 24 well plate. The human mesenchymal stem cell (hMSC) suspension (133,000 cells/mL) was seeded from the top of the scaffolds (300 μL per scaffold, corresponding to approximately 5000 cells/cm^2^). Cells were incubated at 37 °C for 60 min to allow cell attachment. Scaffolds were then flipped, and the same amount of cell suspension was seeded from the top, followed by 60 min incubation at 37 °C. The scaffolds were then transferred to a new non-treated 24 well plate, and 1 mL of fresh growth media was added into each well. The scaffolds were incubated for 7 days in growth media. For osteogenic differentiation studies, growth media was replaced with osteogenic induction media (hMSC osteogenic differentiation medium BulletKit™, Lonza, Basel, Switzerland) at Day 7, and cells were cultured for an additional 14 days. The media was refreshed every 3 days in cell culture studies. 

### 2.6. Cell Culture and Characterization

For stem cell growth studies, the AlamarBlue assay (AlamarBlue™ Cell Viability Reagent, Invitrogen) and PicoGreen assay (Quant-iT™ PicoGreen™ dsDNA Assay Kit, Invitrogen) were used to evaluate the cell proliferation at Days 1, 4, and 7, according to the manufacturer’s protocol. A Tecan plate reader (Infinite M200 Pro, Tecan, Männedorf, Switzerland) was used to complete the assays for these studies. To visualize the attached cells on the scaffolds, cells were washed with DPBS (3×), fixed with 4% formaldehyde for 15 min, followed by DPBS wash (3×), and permeabilization in 0.25% Triton-X DPBS solution for 1 h. Cells were stained for F-actin using rhodamine phalloidin (1:40 in DPBS, Invitrogen). Cell nuclei were stained with 4′,6-diamidino-2-phenylindole (DAPI, 1:2000 in DPBS, Sigma, St. Louis, MO, USA). At Day 7, cells were immunostained for vinculin using the anti-vinculin-FITC antibody (1:50, mouse monoclonal, Sigma). For this purpose, cells were incubated in 10% goat serum (in PBS) for 30 min, washed with staining solution (3×, 3% bovine serum albumin + 0.1% Tween-20 +0.25% Triton-X), and incubated in vinculin antibody in staining solution overnight at 4 °C. Cells were imaged by using a confocal and multiphoton microscopy (TCS SP8 MP, Leica, Wetzlar, Germany).

For differentiation studies, calcium deposition was evaluated at Day 21 by using the alizarin red staining kit (AR, Sigma, St. Louis, MO, USA). After staining was completed, cells were washed with DPBS (3×), and incubated in 10% cetylpyridinium chloride (Sigma, St. Louis, MO, USA) in sodium phosphate buffer (10 mM, pH 7, Sigma) to remove the stain. This solution was then used to quantify calcium content by using a Tecan plate reader (scanned at 405 nm). Alkaline phosphatase activity was studied with the QuantiChrom™ Alkaline Phosphatase Assay Kit (ALP assay Kit, BioAssay Systems, Hayward, NY, USA). Cells cultured within the scaffolds were first lysed with 0.2% Triton-X followed by 3 freeze-thaw circles. The lysate was then reacted with p-nitrophenyl phosphate working solution and scanned at 405 nm using a plate reader (Infinite M200 Pro, Tecan). For osteocalcin (OC) staining, cells were fixed at Day 14 and Day 21. Cells were incubated in 10% goat serum (in PBS) for 30 min, washed with staining solution (3×, 3% bovine serum albumin +0.1% Tween-20 +0.25% Triton-X), and incubated with the OC primary antibody (1:200, monoclonal mouse, Invitrogen) in the staining solution overnight at 4 °C. After removing the antibody containing staining solution and washing the samples with fresh staining solution, cells were incubated in Alexa Fluor 488 rabbit anti-mouse secondary antibody (1:100, Invitrogen) in staining solution for 2 h. Samples were then stained with phalloidin (rhodamine phalloidin, Invitrogen) and DAPI to visualize F-actin and cell nuclei, respectively. Cells were imaged by using a confocal and a multiphoton microscopy (TCS SP8 MP, Leica). All of the collected images were processed using ImageJ (NIH, Bethesda, MD, USA) for further analysis.

### 2.7. Statistics

The data were analyzed using Origin 2016 software. Data are presented as the mean ± the standard deviation. One way ANOVA with Tukey’s HSD post hoc test of means was used to make comparisons between sample groups (*n* ≥ 3 samples per group unless otherwise specified).

## 3. Results

### 3.1. 3D Printing of PCL Scaffolds

**Figure 2:**
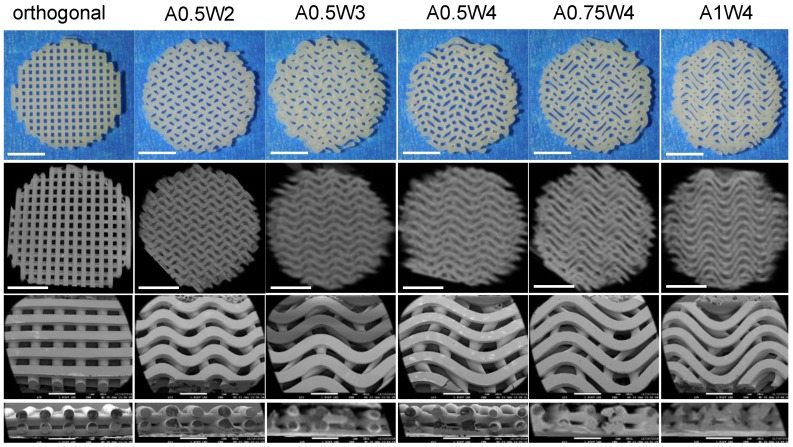
Images of the scaffolds. From the top to bottom row, images correspond to pictures (top view), micro-computed tomography (micro-CT) images (top view), scanning electron microscope (SEM) images, and SEM cross-section images. Scale bars are 500 microns for pictures and micro-CT images and 1 mm for SEM images.

PCL scaffolds with six distinct designs, including one linear design (orthogonal) and five wavy designs in the form of a sinusoidal wave with varying amplitude (A) and wavelength (W) (A0.5W2, A0.5W3, A0.5W4, A0.75W4, and A1W4, where the numbers following A and W denote the actual values of A and W in mm) were printed (Table 1). Figure 2 shows the pictures, micro-CT images, and SEM images of the scaffolds. SEM images were used to measure the printed strut width and spacing between struts for each design, and the results are summarized in Table 1. Briefly, when all the designs were considered, the average strut width was within the range of 460 ± 58 to 533 ± 9 μm, and the spacing between struts (strut-to-strut distance) was within the range of 277 ± 59 to 395 ± 6 μm. 

### 3.2. Mechanical Tests

**Figure 3:**
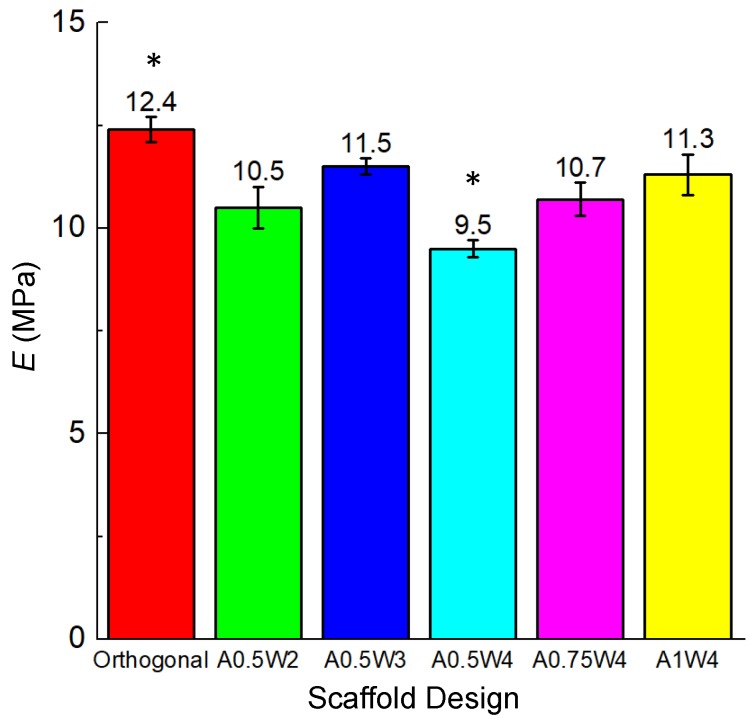
Young’s modulus (E) values of the scaffolds for each scaffold design. * *p* < 0.005 for orthogonal vs. A05W2, A0.5W4, and A1W4; and for A0.5W4 vs. all sample groups.

Compression tests were performed on each sample group, and the results are summarized in Table 1 and Figure 3. The compressive modulus (Young’s modulus, E) of all the designs was in the range of 9.5–12.4 MPa (Table 1). E (9.5 MPa) for the A0.5W4 design (with the highest porosity, ~62%) was significantly lower than the rest of the sample groups. The orthogonal design (E = 12.4 MPa and porosity = ~56%) showed significantly higher E as compared to A0.5W2 (E = 10.5 MPa and porosity = ~56%), A0.5W4, and A1W4 (E = 11.3 MPa and porosity = ~57%).

### 3.3. Growth Study 

**Figure 4:**
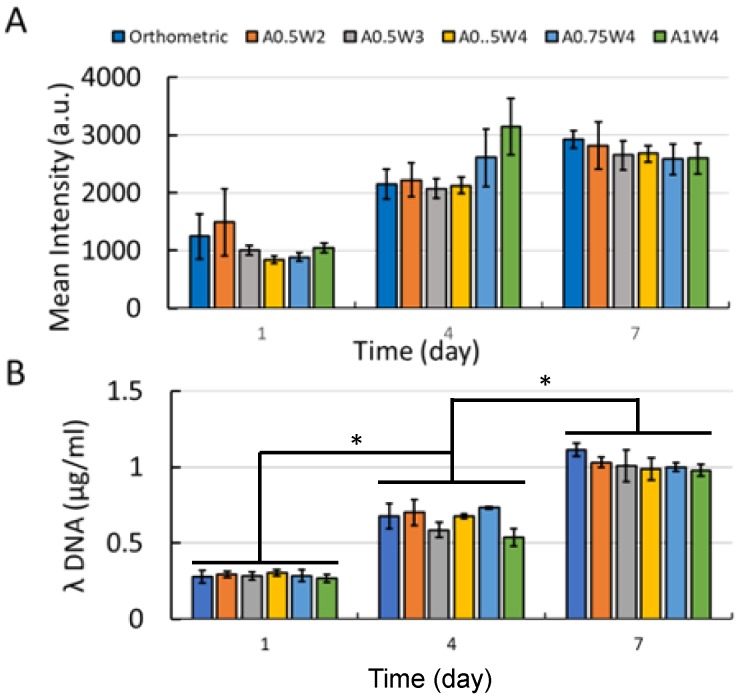
(**A**) AlamarBlue assay results; (**B**) PicoGreen assay results. (* *p* < 0.005).

**Figure 5:**
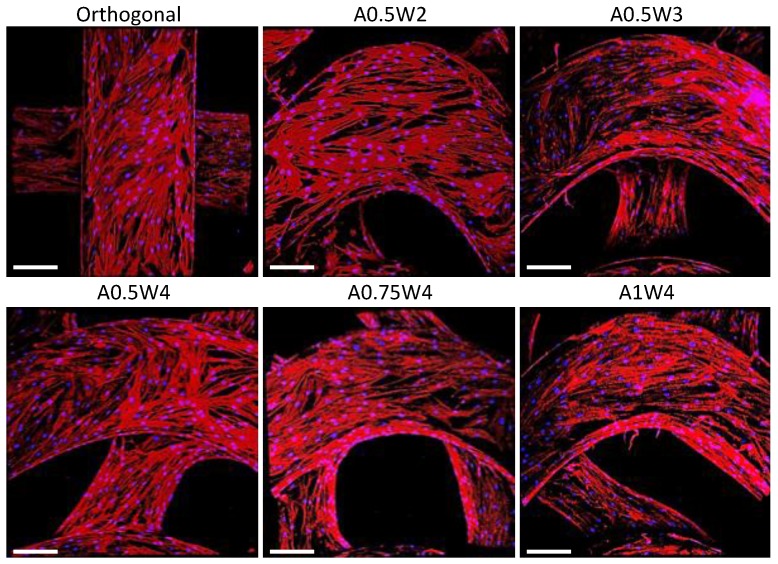
Multiphoton confocal images of the human mesenchymal stem cells (hMSCs) cultured on the scaffolds for seven days. Cells were stained for F-actin (red) and nuclei (blue). Scale bars are 200 microns.

The hMSC growth studies were performed by culturing cells in growth media for up to seven days. The results for AlamarBlue assay and PicoGreen assay are shown in Figure 4. The AlamarBlue assay results showed that the measured mean intensities increased from Day 1 to Day 7, which indicated an increased metabolic activity with culture time. There was an exception for A1W4, which showed a drop from Day 4 to Day 7. At Day 7, no significant difference was observed between the test groups. For the PicoGreen assay, a similar trend was observed as the mean value of λ-DNA ascended from Day 1 to Day 7. At Day 7, there was no difference between the test groups. The multiphoton confocal images of the stem cells (F-actin in green and cell nuclei in blue) cultured on the 3D printed scaffolds for seven days are given in Figure 5. F-actin filaments were aligned with the printed struts that formed the scaffolds, and this alignment was more pronounced in the curved regions in wavy scaffolds.

### 3.4. Differentiation Study 

**Figure 6:**
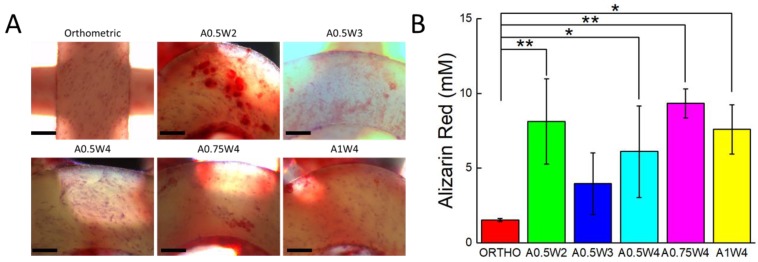
(**A**) Optical microscopy images of the hMSCs stained for alizarin red (red) after culture in osteogenic induction media for 21 days. Scale bars are 200 microns. (**B**) Alizarin red concentration indicating calcium deposition at Day 21. (* *p* < 0.15, ** *p* < 0.05, for *n* = 3).

**Figure 7:**
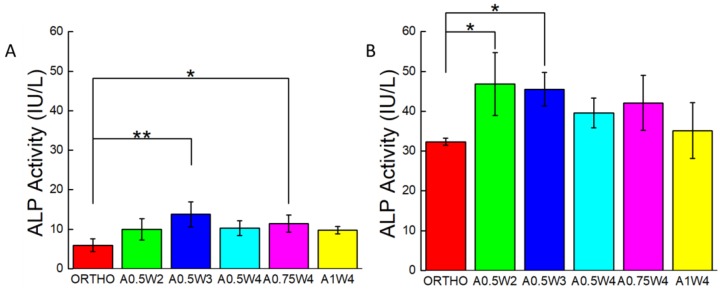
Alkaline Phosphatase (ALP) activity assay results for: (**A**) Day 14 and (**B**) Day 21 (* *p* < 0.15, ** *p* < 0.05, for *n* = 3).

**Figure 8:**
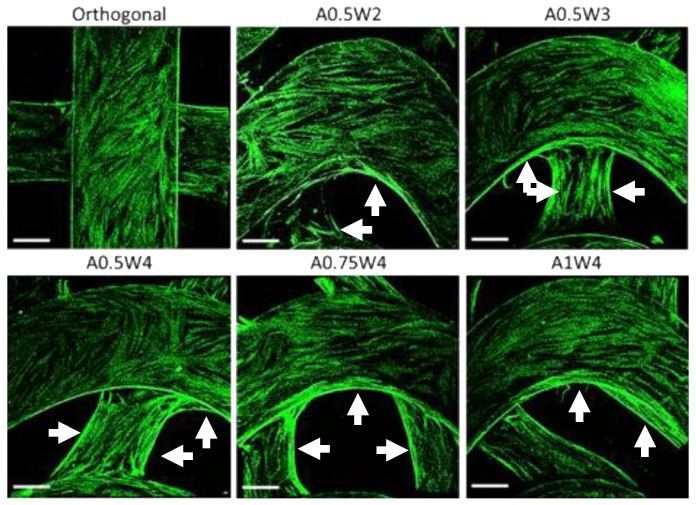
Multiphoton confocal images of hMSCs that are stained for vinculin (green) at Day 7.

**Figure 9:**
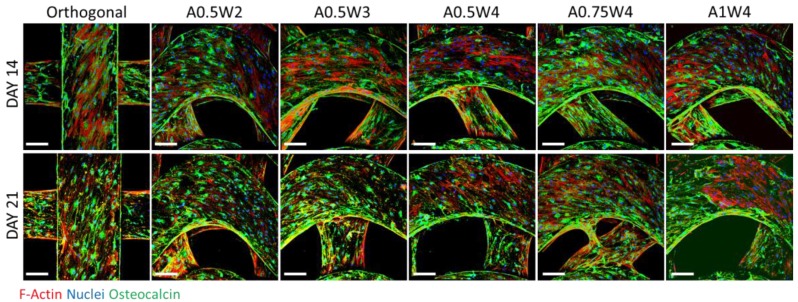
Multiphoton confocal images for hMSCs that were cultured in osteogenic induction media for 14 (**top** row) and 21 days (**bottom** row). Cells were immunostained for osteocalcin (green) and stained for F-actin (red) and cell nuclei (blue). Scale bars are 200 microns.

Osteogenic differentiation of hMSCs cultured on the 3D printed scaffolds was studied for up to 21 days. Figure 6 shows the results from alizarin red (AR) staining and assay. The scaffolds with wavy designs showed more staining (Figure 6A) and higher values of mean calcium deposition (Figure 6B). The value of the mean calcium deposition in wavy groups was in the range of 2.5 to six times higher than that of the orthogonal group. Specifically, the average calcium deposition was equal to 9.33 ± 0.98 mM for A0.75W4, 8.14 ± 2.86 mM for A0.5W2, 7.60 ± 1.65 mM for A1W4, 6.12 ± 3.07 mM for A0.5W4, 3.96 ± 2.06 mM for A0.5W3, and 1.53 ± 0.10 mM for orthogonal scaffolds, in descending order. ALP activity assay results, at Culture Days 14 and 21, are given in Figure 7. Our results showed an increase in ALP activity for all sample groups from Day 14 to Day 21, and the ALP activity of the wavy scaffolds was higher than that of the orthogonal group at both Day 14 and Day 21 (Figure 7). At Day 14, A0.5W3 (13.16 ± 3.17 a.u.) was significantly higher than the orthogonal group (5.96 ± 1.58 a.u.). At Day 21, A0.5W2 (46.83 ± 7.90 a.u.) and A0.5W3 (45.51 ± 4.20 a.u.) were much higher than that of the orthogonal group (32.31 ± 0.89 a.u.). Representative fluorescent images showing vinculin staining at Day 7 are shown in Figure 8. We observed more pronounced vinculin fibers that were aligned with the wavy struts for wavy scaffolds as compared to diffused and randomly oriented vinculin for the orthogonal scaffold. Figure 9 shows the representative confocal images of the hMSCs cultured on 3D printed scaffolds, in which cells were stained for osteocalcin (OC, green), F-actin (red), and nuclei (blue) at Culture Day 14 and 21. Osteocalcin staining was more pronounced on curved struts as compared to linear struts. 

## 4. Discussion

In this study, we used extrusion based DIW printing technology to fabricate PCL scaffolds. DIW allowed us to 3D print scaffolds directly from PCL pellets, which were melted within and extruded from a steel syringe attached to the print head. PCL was selected as a model polymer as it is a “Generally Recognized As Safe” (GRAS) polymer by the U.S. Food and Drug Administration and widely used to 3D print tissue engineering scaffolds for both in vitro and in vivo studies [27,35,75]. We used human adult mesenchymal stem cells (hMSCs) as the main cell line due to their ability to proliferate, migrate, and differentiate into a wide range of tissue specific phenotypes including bone, cartilage, and muscle. Stem cells are known to feel and respond to their microenvironment (matrix stiffness, topography, and bioactivity) by regulating their behavior [56,60]. Here, we focused on the topography, or scaffold architecture. To investigate the effects of 3D scaffold architecture on stem cell osteogenesis, we constructed scaffolds using struts in sinusoidal waveforms, systematically varying the amplitude and the wavelength (Figure 1 and Figure 2, Table 1). The sinusoidal waveform design created highly curved strut surfaces forming 3D scaffolds with wavy patterns. Our motivation to create wavy scaffolds was based on previous studies, which clearly showed the importance of substrate curvature on stem cell osteogenesis [69,70]. 

The minimum wavelength and amplitude achievable for a strut size around 500 μm were 2 mm and 0.5 mm (A0.5W2). While keeping the amplitude constant at 0.5 mm, the wavelength was increased to 3 mm (A0.5W3) and 4 mm (A0.5W4). For the 4 mm wavelength, the amplitude was increased to 0.75 mm (A0.75W4) and 1 mm (A1W4). These geometrical constraints allowed us to create scaffolds with an average strut-to-strut distance of approximately 350 μm (Figure 2, Table 1). Note that the effect of pore size in bone scaffolds has been well studied [15,24,76,77,78,79,80,81], and a minimum pore size of ~150 μm is usually required for cell migration and tissue ingrowth [3,82,83,84]. We then investigated the effect of scaffold design on mechanical properties of the scaffolds (Figure 3). The compressive modulus (*E*) values were determined by the design, i.e., strut-to-strut contacts between layers, and the overall scaffold porosity. *E* values were significantly the highest for orthogonal scaffolds (12.5 MPa) mainly because these scaffolds inherently displayed more strut-to-strut contacts, considering that this design had 16 struts per layer, whereas all of the wavy designs had 15 struts per layer. This design also had one of the lowest porosities with ~56%. When wavy scaffolds were compared, A0.5W4 showed the significantly highest porosity (~62%) corresponding to the significantly lowest *E* value of 9.5 MPa followed by A0.75W4 (58%, 10.7 MPa), A1W4 (57% 11.3MPa), and A0.5W3 (56%, 11.5 MPa). A0.5W2 (56%, 10.5 MPa) was an exception and did not follow the trend. This was due to reduced strut-to-strut contacts due to the design (Figure 2). Although the overall scaffold modulus determines the mechanical support level that a scaffold can provide when implanted, it did not affect the stem cell behavior in our study. This is because the stem cells feel the mechanics of the individual struts (which was uniform for all scaffold groups) that they reside on when seeded on to the scaffolds [74].

First, the growth study was conducted to determine the attachment and proliferation of the hMSCs cultured on our scaffolds. The metabolic activities of the cells were not significantly different from each other at each culture day, but increased significantly with culture day, reaching a maximum at Day 7 (Figure 4A). The same trend was observed when the DNA was quantified (Figure 4B). Note that this trend was not true for the A1W4 and A0.75W4 sample groups, for which the metabolic activity reached a maximum at Day 4 and did not change significantly at Day 7. Yet, the DNA count did not show this unexpected trend for these two sample groups, which represented the cell proliferation more accurately. F-actin staining at Day 7 confirmed that cells attached onto the struts and formed confluent layers at Day 7, taking the shape of the struts. Cells on wavy scaffolds were highly elongated, especially on the curved edges with well-defined F-actin filaments aligned with the scaffold curvature as compared to much bulkier cells on orthogonal scaffolds (Figure 5). In addition, stem cells on wavy scaffolds showed mature vinculin (focal adhesion marker) patches as compared to diffused vinculin staining of cells on orthogonal scaffolds at Day 7 (Figure 8). Focal adhesion is a vital step in osteogenesis [85] in which vinculin directs the interaction between talin and actin to direct the focal adhesion process [86]. We investigated if these significant changes in stem cell morphology, F-actin expression, and focal adhesion on wavy scaffolds as compare to orthogonal scaffolds correlated with stem cell osteogenesis on wavy scaffolds. It was also noted that the curvature had a direct effect on cell proliferation, and studies have shown that curvature induced contractility enhances proliferation and cell growth [87,88,89]. In our study, we did not observe a significant difference in proliferation between sample groups. This was not contradictory to the literature as each of our wavy scaffolds displayed both concave and convex curvature, and the overall cellular behavior was collective rather than distinct for each type of curvature. 

The differentiation studies were conducted after the cells reached a confluent state at Day 7, as shown by the growth studies (Figure 4 and Figure 5). At Day 7, the growth media was replaced with osteogenic induction media, and cells were cultured for 14 additional days in induction media, a total of 21 days in culture. To assess the osteogenic differentiation of hMSCs, we quantified calcium deposition and ALP activity and performed immunostaining for osteocalcin. The AR assay was used to probe the deposition of calcium. Optical microscope images revealed that wavy scaffolds showed more stained regions than the orthogonal group. When quantified, all the wavy scaffolds showed higher calcium deposition than the orthogonal group, and in particular, two groups (A0.5W2 and A0.75W4) showed significantly higher calcium deposition (Figure 6). These results indicated that the overall contribution of the curvature on these two scaffolds on cellular contractility induced calcium deposition was the highest. ALP is a well known biological marker for stem cell osteogenesis [90]. ALP activity increased significantly for all of the scaffold groups from Day 14 to Day 21 (Figure 7). All the wavy groups showed higher ALP activity than the orthogonal group. However, the differences between wavy groups and the orthogonal group were not as significant as the results from the AR assay. This could be because the ALP expressed at earlier stages of the osteogenesis process. At Day 14, A0.5W3 showed significantly higher ALP activity (*p* < 0.05) when compared to the orthogonal group. At Day 21, both A0.5W2 and A0.5W3 were substantially higher than the orthogonal group (*p* < 0.15). To supplement our quantitative differentiation assays, we performed OC immunostaining (Figure 9) as a marker for osteogenesis. Qualitatively, we observed increasing OC staining with culture day, and wavy scaffolds showed more OC staining, in particular in the curved regions of the scaffolds. The enhanced osteogenesis behavior on wavy scaffolds could be explained as the effect of the curvature, which led to a highly aligned and stretched cellular morphology (Figure 5) with mature focal adhesions (Figure 8), leading to highly contractile cells promoting osteogenesis. We strongly believe that our results clearly showed the importance of scaffold architecture on hMSC osteogenesis and would help to develop novel scaffold architectures for bone tissue regeneration.

## 5. Conclusions

In this study, we developed 3D printed PCL scaffolds with wavy or linear patterns to investigate the effects of a wavy scaffold architecture on the osteogenic differentiation of hMSCs. When cultured in growth media, hMSCs attached and proliferated, forming confluent layers on the scaffolds within seven days. We found that hMSCs spread by taking the shape of the curved surfaces and exhibited elongated F-actin filaments and mature focal adhesion sites (vinculin staining). In contrast, hMSCs were bulkier in shape and showed dispersed vinculin staining on the orthogonal scaffold. We found that hMSCs showed significantly higher calcium deposition, higher ALP activity, and significantly pronounced osteocalcin staining when cultured on wavy scaffolds as compared to orthogonal scaffolds. These results are important in that they clearly showed the importance of scaffold architecture on hMSC osteogenesis and may provide guidance on novel bone scaffold/graft design for pre-clinical and clinical applications.

## Figures and Tables

**Table 1:** Design and printing parameters for the scaffolds.

Parameter	Orthogonal	A05W2	A0.5W3	A0.5W4	A0.75W4	A1W4
Amplitude (mm)	-	0.5	0.5	0.5	0.75	1
Wavelength (mm)	-	2	3	4	4	4
Strut diameter (μm)	533 ± 9	497 ± 73	490 ± 36	513 ± 30	510 ± 32	460 ± 58
Strut spacing ^1^ (μm)	395 ± 6	277 ± 59	350 ± 53	396 ± 72	308 ± 72	336 ± 103
Struts per layer	16	15	15	15	15	15
Temperature ^2^ (°C)	80	80	80	80	80	80
Print pressure (Pa)	7 × 10^5^	7 × 10^5^	7 × 10^5^	7 × 10^5^	7 × 10^5^	7 × 10^5^
Print speed (mm/s)	4	4	6	5	5	5
*E* ^3^ (MPa)	12.4 ± 0.3	10.5 ± 0.5	11.5 ± 0.2	9.5 ± 0.2	10.7 ± 0.2	11.3 ± 0.5
Porosity ^4^ (%)	56.3 ± 0.7	56.5 ± 1.2	55.9 ± 0.3	61.7 ± 0.9	57.6 ± 0.5	57.2 ± 3.1

^1^ Strut-to-strut distance. ^2^ Print temperature. ^3^ Young’s modulus from compression tests. ^4^ Micro-CT results.
